# Difference between Minorities and Majorities in the Association between COVID-19-Related Stress and Psychological Distress: A Socio-Ecological Perspective and the Moderating Role of Parenthood

**DOI:** 10.3390/ijerph19148283

**Published:** 2022-07-07

**Authors:** Lubna Tannous-Haddad, Dorit Hadar-Shoval, Michal Alon-Tirosh, Kfir Asraf, Orna Tzischinsky

**Affiliations:** 1 Behavioral Sciences Department, The Max Stern Yezreel Valley College, Yezreel Valley 1930600, Israel; lubnah@yvc.ac.il (L.T.-H.); michalt@yvc.ac.il (M.A.-T.); 2 Psychology Department, The Max Stern Yezreel Valley College, Yezreel Valley 1930600, Israel; kfira@yvc.ac.il; 3 Educational Counseling Department, The Max Stern Yezreel Valley College, Yezreel Valley 1930600, Israel; orna@yvc.ac.il

**Keywords:** COVID-19-related stress, psychological distress, socio-ecological model, parenthood, minorities and majorities, Jews and Palestinians

## Abstract

This study introduces a socio-ecological perspective of differences in psychological distress between the Palestinian minority and Jewish majority citizens of Israel during lockdown due to COVID-19. The study examines the association between COVID-19-related stress and psychological distress, and the moderating effect of parenthood. Online questionnaires, completed by 1934 participants (1391 Jews, 552 Palestinians; 1306 parents, 637 without children; 54.86% female, 45.13% male; M age = 40.38, SD = 13.77) assessed COVID-19-related stressors and depression, anxiety, and stress. The Palestinian minority showed a higher level of COVID-19-related stress and psychological distress than the Jewish majority. Parenthood showed a moderating effect on the association between COVID-19-related stress and distress for the Jewish majority but not the Palestinian minority. The results emphasize the significance of considering social status when seeking to understand the differences between minorities and majorities in terms of distress and resilience during pandemic events, and the need for cultural sensitivity and awareness when issuing instructions in such circumstances. Additionally, the results highlight the potential role of parenthood as a resilience factor, depending upon social status.

## 1. Introduction

Coronavirus disease 2019 (COVID-19) has had a multi-level impact across many domains, e.g., health, economic, social, and personal. As people all over the world cope with mandatory quarantines, economic recessions, and the threat to their health, the effect of these circumstances on their psychological wellbeing is of real concern, as discussed by both cross-sectional and longitudinal studies [1,2,3,4,5]. COVID-19-related stress can be triggered by COVID-19-related circumstances such as financial and health-related issues, social isolation, and difficulty obtaining essential supplies, as well as persistent worries regarding oneself or family members becoming ill [6,7,8,9]. Minorities may experience these effects more intensely, as there are usually economic, social, and educational disparities between minority and majority groups [10,11,12]. Iob et al. found that, during the first month of the COVID-19 outbreak in England, a higher frequency of behaviors associated with distress, such as self-harm and suicide attempts, were reported among Black, Asian, and other ethnic minorities compared with those in the majority groups [13]. Meanwhile, a study conducted in the general US population during the pandemic found being Hispanic to be associated with anxiety and depressive symptoms [14]. In Israel, members of the Palestinian minority were found to exhibit more distress during the COVID-19 pandemic compared with members of the Jewish majority [15]. Therefore, when considering general public health, minorities call for special attention.

The current study offers an explanation for the differences in the mental health of minorities compared with majorities in terms of the socio-ecological perspective by studying the case of the Palestinian minority and the Jewish majority in Israel. It also seeks to determine whether, for these two ethnic groups, parenthood acts as a resilience factor for psychological distress. The study’s novelty lies in the introduction of the ecological model as a framework for conceptualizing the differences between minorities and majorities, and in suggesting the mechanism behind parenthood as a resilience factor.

### 1.1. Socio-Ecological Perspective

Bronfenbrenner’s socio-ecological model posits that behaviors are shaped by the immediate environment, known as the “microsystem” (e.g., family and friends); relationships between the immediate environment and society, known as the “exosystem” (e.g., family status and social support); and more distal environmental factors, known as the “macrosystem” (e.g., cultural practices and governmental policies) [16]. In times of adversity, a person’s resilience capital is influenced by the support obtained from these various systems [17]. Different populations vary in their patterns of reliance on the systems, a difference that can affect their health. The Palestinian minority and the Jewish majority in Israel demonstrate these differences.

### 1.2. The Palestinian Minority and the Jewish Majority

Israeli society is divided into two ethnic groups: the Palestinian minority (representing 20% of the Israeli population) and the Jewish majority (representing 80% of the Israeli population). The behavior of the Palestinians (commonly referred to as Arabs of Israel), as a minority in Israel, is highly influenced by two major factors: their collective culture and their historical narrative. Palestinian society is collective in nature, which, among other things, means that family (parents, children, grandparents, cousins, uncles, aunts, etc.) and the community play a central role in people’s lives, including involvement in major personal decisions and choices. It is also believed that individuals facing obstacles and problems should resolve them within the family, without seeking external help or advice. Despite the recent progress of Israel’s Palestinian society toward modernization, the relationships between individuals and their families remain very strong and are based on mutual dependence. Members are expected to turn to their families for assistance in any social, economic, or health-related difficulties [18,19].

In addition, the Palestinians in Israel have an ambiguous relationship with the state because of their shared history and the ongoing Israeli–Palestinian conflict [20]. According to the Palestinian historical narrative, the declaration of independence of the state of Israel in 1948 was preceded by a massive forced deportation of the original Palestinian population to other countries. “Nakba”, meaning “catastrophe”, is the term used to describe the experience of losing homes and land [20]. Today, the minority population that remained on its land lives mostly in dispersed villages and small cities, and has preserved its ethnic identity [21]. On the one hand, they are Israeli citizens; on the other hand, there is a feeling of mistrust toward the Israeli government and policies, as this minority group experiences inequalities in terms of economic, cultural, and service aspects [22,23,24,25]. The literature has illustrated a higher prevalence of emotional distress and health problems among the Palestinian minority over the years [26,27].

In contrast, naturally, most of the Jewish majority identifies with the Israeli government and its policies, and feels that the state represents them. Therefore, in times of need, the Jewish Israeli citizens are more likely, compared with the Palestinians, to seek various official institutions as a source of support, reinforcing the support received from their family members [13,28]. From Bronfenbrenner’s socio-ecological model perspective, Palestinians and Jews in Israel have different patterns of reliance on the various systems. The microsystem and the exosystem, meaning the family and community, respectively, play a major role in the lives of Palestinians because, as a minority, they mistrust and feel less supported by the macrosystem represented by the government and its policies [22]. The Jewish majority, in contrast, can rely on all the three systems. A concrete example of this can be seen in the differences between Palestinians’ and Jews’ willingness to be vaccinated against COVID-19 [24,25].

During the COVID-19 pandemic, a social distancing policy was implemented in Israel. Israeli citizens were asked to stay at home and were only allowed to associate with people from the same household [29]. These measures restricted social contact with family and friends. From a socio-ecological perspective, support from the microsystem and the mesosystem was negatively impacted, while the exosystem became dominant (as governmental policies affected all areas of life). For the Palestinian minority, this situation reduced the available support and resilience capital. A study that compared Jewish and Palestinian citizens of Israel in terms of the levels of distress experienced during the pandemic found that Palestinians exhibited more distress than Jews [15].

### 1.3. Parenthood and Distress

Another difference between the two societies during the COVID-19 pandemic relates to the influence of parenthood on distress. A previous study found that Jews who had children reported less distress than those who did not [15]; a similar positive influence of parenthood on distress during the pandemic was found in a sample from the Philippines [30]. However, among the Palestinian minority in Israel, parenthood was not found to influence distress levels [15]. These findings raise questions about the mechanism by which parenthood acts as a resilience factor for majority groups but not for minority groups.

Parenthood can either promote well-being or cause distress, depending on various factors, such as social support [31]. Parents who receive high social support experience increased positive emotions and decreased negative emotions, which is associated with reduced distress [32]. Social support can be obtained through various ecological systems, and more support systems predict better wellbeing and higher resilience capital [33]. In the Israeli context, because of the social distancing policy implemented during the pandemic, the Palestinian minority had fewer support systems than the Jewish majority (this was because they were not allowed to socialize with family members who did not live in the same house, which minimized their main source of social support, and they did not identify with the support from the government, unlike the Jewish citizens). Accordingly, it can be expected that, while parenthood acted as a resilience factor for distress among the Jewish majority, it did not have the same effect for the Palestinian minority.

### 1.4. The Current Study

The current study offers a socio-ecological perspective on the differences in mental health between minorities and majorities. Specifically, it explores the differences between the Jewish majority and the Palestinian minority in Israel, in terms of mental health during COVID-19, as well as the role of parenthood as a resilience factor for these two ethnic groups.

The study had two hypotheses: First, there is a difference between the Palestinian minority and the Jewish majority regarding the levels of COVID-19-related stress and psychological distress experienced during the pandemic, with Palestinians reporting higher stress and distress than Jews. The second hypothesis was that parenthood moderates the association between COVID-19-related stress and psychological distress for the Jewish majority but not for the Palestinian minority.

## 2. Materials and Methods

### 2.1. Participants and Data Collection

The current study was based on data collected by the authors through a cross-sectional survey that was conducted in Israel approximately 2 months after the implementation of the initial pandemic-related restrictions. The survey was designed to assess the public’s demographic characteristics and their immediate psychological and behavioral responses to the COVID-19 pandemic, and was conducted via an anonymous online questionnaire that was created using Qualtrics (https://www.qualtrics.com). The survey was sent to participants online through iPanel (https://www.ipanel.co.il), a large Israeli panel service. The study protocol was approved by the College Institutional Review Board (2020-54 YVC EMEK). Questionnaire completion was voluntary, and respondents were informed that they could cease their participation at any point. Completed questionnaires were excluded from the final analysis if the respondents had failed attention checks, if they had completed the measures in less than 10 min, and if they had provided implausible responses (for example, they chose the same answer throughout the questionnaire). Consequently, data from 1943 participants were advanced to the final analysis

The participants for the present study comprised 1943 adults, who were recruited online using the methods described below. The inclusion criteria were being aged between 20 and 75 years and possessing literacy in at least one of the languages in which the survey was administered (Hebrew or Arabic). There were no exclusion criteria. The sample comprised 1391 Jews (71.6%) and 552 Palestinians (28.4%); most of the participants (67.2%) were parents (i.e., they had children) and over half of the sample (54.8%) were female. The participants’ mean age was 40.3 years (standard deviation (SD) = 13.7), with the mean years of education being 14.6 (SD = 2.5). The majority of the participants lived in urban areas (75.1%), were currently employed (62.1%), and had experienced a deterioration in their economic status since the beginning of the COVID-19 pandemic (64.4%). The participants’ demographic data are shown in Table 1.

### 2.2. Measures

#### 2.2.1. Demographic Information

The questionnaire included a section devoted to demographic characteristics, which comprised items on ethnicity, parenthood, gender, age, area of residence, and education level. Additional items concerned the influence of the COVID-19 pandemic on the participants’ employment and economic status.

#### 2.2.2. COVID-19-Related Stress

The COVID-19-related stress scale [8] comprises 13 COVID-19-related stressors, including financial problems, inability to spend time with friends or family, changes to normal routines, cancellation of travel plans, challenges at home, trouble obtaining essential supplies or services, hearing distressing news reports, uncertainty about one’s self or others becoming infected with COVID-19, difficulty meeting work or educational responsibilities, increased work and/or family responsibilities, and uncertainty regarding the future. Using a scale ranging from 1 (“not at all stressful”) to 4 (“very stressful”), the participants were asked to rate how stressful they had found each of these 13 stressors since the implementation of the pandemic-related restrictions. For each participant, the total score for COVID-19-related stressors was computed by summing the scores for each of the 13 items; higher scores indicated greater stress. The internal consistency (Cronbach’s alpha) was 0.87.

#### 2.2.3. Depression, Anxiety, and Stress Scale

The Depression, Anxiety, and Stress Scale (DASS-21; [34]; Hebrew version, retrieved from the DASS-21 website: http://www2.psy.unsw.edu.au/dass/, accessed on 20 March 2020) includes 21 items, evaluating depression (seven items), anxiety (seven items), and stress (seven items). All items are scored using a four-point Likert scale ranging from 0 (“never”) to 3 (“most of the time”). A score above 11 on the depression scale indicates severe depression, a score above 8 on the anxiety scale indicates severe anxiety, and a score above 9 on the stress scale indicates moderate or severe stress. The DASS-21 demonstrates good reliability and validity among both clinical and non-clinical samples [35]. In the current study, the internal reliability (Cronbach’s alpha) was 0.97 for the total score, 0.90 for depression, 0.85 for anxiety, and 0.90 for stress.

### 2.3. Data Analysis

Hypothesis 1 was analyzed using independent sample *t*-tests, with bias-corrected and accelerated bootstrapped confidence intervals (CIs; 5000 samples) and Hedges’ *g* as effect size estimators. Hypothesis 2 was analyzed through moderation analysis using Model 3 of Hayes’ PROCESS macro (v 3.5) for SPSS [36]. The predictors of the moderation analysis were COVID-19-related stress, ethnicity (Palestinian/Jewish), and parenthood (having children/not having children). Age, years of education, gender (male/female), area of residence (urban/rural), the effect of COVID-19 on employment status (still working/not working), and the effect of COVID-19 on economic status (income affected/income not affected) were included in the model as covariates. The three two-way interaction terms and the three-way interaction term were also included in the analysis. Continuous variables (COVID-19-related stressors) were centered. The statistical significance of the effects in the moderation analysis was determined using bootstrapped CIs. Data that deviated by more than three SDs from the mean were excluded from the analyses.

## 3. Results

### 3.1. Cohort Description

The Palestinian group was younger (Palestinians: M = 32.86 years, SD = 9.68; Jews: M = 43.33 years, SD = 14.01), included more females (Palestinians: 65.39%; Jews: 50.68%), was less urban-based (Palestinians: 56.52%; Jews: 82.53%), was more economically affected by the pandemic (Palestinians: 73.18%; Jews: 60.96%), and had a lower proportion of members who were still working (Palestinians: 52.53%; Jews: 65.92%). These variables were entered as covariates into the moderation model. In addition, the Palestinian group had a lower proportion of participants with children (Palestinians: 53.44%; Jews: 72.68%).

### 3.2. The Difference between the Palestinian Minority and the Jewish Majority Regarding the Levels of COVID-19-Related Stress and Psychological Distress Experienced during the Pandemic

We hypothesized that there would be a difference between the Palestinian minority and the Jewish majority in terms of psychological distress (measured using the DASS-21) and COVID-19-related stress, with Palestinians reporting higher levels of both when compared with Jews. As hypothesized, there was a significant difference between the groups regarding distress (t (1932) = −13.55, *p* < 0.001, CI = −11.17, −8.34, Hedges’ *g* = 0.83): the Palestinian group (*n* = 552; M = 18.51, SD = 15.87) had a higher mean distress score than the Jewish group (*n* = 1382; M = 8.74, SD = 9.60). There was also a significant difference between the groups in terms of COVID-19-related stress (t (1932) = −12.92, *p* < 0.001, CI = −6.27, −4.60, Hedges’ *g* = 0.69): the Palestinian group (M = 36.60, SD = 8.62) had a higher mean COVID-19-related stress score than the Jewish group (M = 31.17, SD = 7.43). The differences between the groups were underlined by the effect sizes for the comparisons.

### 3.3. Parenthood as a Moderator of the Association between COVID-19-Related Stress and Psychological Distress

We hypothesized that, for the Jewish majority, the association between COVID-19-related stress and psychological distress (as measured using the DASS-21) was moderated by parenthood, but parenthood had no such effect for the Palestinian minority. To examine this hypothesis, we conducted a moderation analysis. Prior to the moderation analysis, we examined the zero-order correlation between the COVID-19-related stress scale and the DASS-21, as they both feature a stress component. The Pearson correlation was significant (*r* (1932) = 0.45, *p* < 0.001, R^2^ = 20.59%); however, the strength of the association was medium, suggesting that the two variables do not measure the same trait and can be entered into a model together. The moderation model was significant (*F*(12, 1892) = 66.50, *p* < 0.001) and explained 29.67% of the variance (R^2^). The model is summarized in Table 2. Table 2 shows that COVID-19-related stress was significantly and positively associated with the DASS-21 score. Being a Palestinian was significantly associated with higher DASS-21 scores, and having children was significantly associated with lower DASS-21 scores. The highest-order interaction term, the three-way interaction of ethnicity × parenthood × COVID-19-related stress, was statistically significant and added 0.26% to the explained variance, even after controlling for the covariates.

Probing the interaction, we considered the parenthood × COVID-19-related stress interaction on DASS-21 scores separately for the Jewish and Palestinian groups. While the parenthood × COVID-19-related stress interaction was not significant for the Palestinian group (B = 0.09, *F*(1, 1892) = 3.27, *p* = 0.070), the interaction was significant for the Jewish group (B = −0.08, *F*(1, 1892) = 3.90, *p* = 0.048.) Therefore, for Jews, parenthood acts a moderator in the association between COVID-19-related stress and DASS-21 scores.

**Figure 1:**
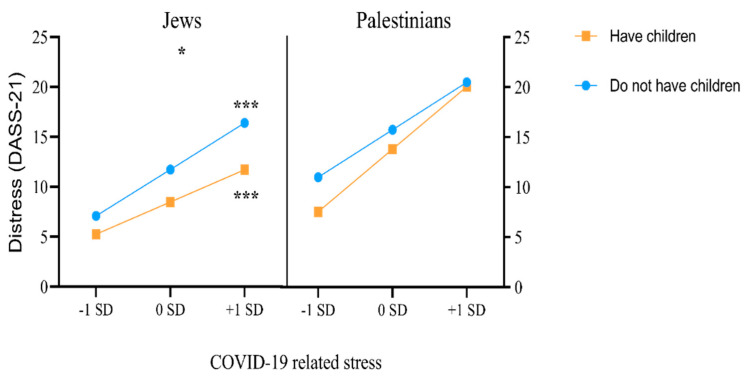
Differences between the Palestinian minority and the Jewish majority for the moderating effect of parenthood on the association between COVID-19-related stress and distress. *** *p* < 0.001, * *p* < 0.05. DASS-21: Depression, Anxiety, and Stress Scale.

In the Jewish group, the association between COVID-19-related stress and DASS-21 scores was significant for both the participants who did not have children (B = 0.57, t = 7.39, *p* < 0.001) and the participants who had children (B = 0.39, t = 8.91, *p* < 0.001). However, the association was significantly stronger for Jews who did not have children (Figure 1). The finding that parenthood has a mitigating effect was supported when we compared the mean scores of the DASS-21 for the Jews with and without children, and who had a high level of COVID-19-related stress (+1 SD condition): the Jews who had children had a lower mean DASS-21 score (mean difference = 4.67) than those without children.

## 4. Discussion

The current study offers an explanation for the differences in the mental health of minorities compared with majorities during the COVID-19 pandemic in terms of the socio-ecological perspective by studying the case of the Palestinian minority and Jewish majority in Israel. The study compared the two populations’ mental health in terms of the levels of COVID-19-related stress and psychological distress. It also provided more insights about the role of parenthood as a moderator of the association between these two variables during the COVID-19 pandemic.

The study found a substantially higher level of both COVID-19-related stress and psychological distress among the Palestinian minority compared with the Jewish majority. These findings accord with those of existing research on the psychological repercussions of COVID-19 worldwide. Previous studies have reported that minorities experience higher levels of distress than majorities [14,15]. The present findings are also consistent with those of previous studies that observed differences in psychological distress between the Palestinian minority and the Jewish majority in Israel in relation to other contexts. Gelkopf et al. examined Israeli citizens at a time when Israel had been experiencing terror attacks for 44 months and observed much lower resilience among Palestinian citizens than among Jewish citizens [37]; furthermore, Kimhi et al. reported that Palestinian students experience a higher level of distress than Jewish students in relation to the ongoing Palestinian–Israeli conflict [38].

During the pandemic, quarantines and social distancing regulations were imposed, meaning most citizens of Israel were isolated from their family members and local communities. Thus, from a socio-ecological perspective, the microsystem and mesosystem were unavailable. However, the Jews, who mostly identify with the state, could still rely on the exosystem represented by the government and its policies. On the other hand, Palestinians, who do not tend to trust the state’s policies, did not rely on it and did not feel that they had the same level of support available. From the Palestinian perspective, the social distancing instructions have had two major implications. First, the instructions ordered them to stay away from their most important support source and targeted an essential component of their collective culture; second, the instructions were given by the government of Israel, which they do not trust. These differences between the Palestinian minority and the Jewish majority had an impact on how these groups perceived the pandemic-related instructions and reacted to the situation. This is particularly evident in the Palestinians’ difficulty in cooperating with being vaccinated against COVID-19 [25] and in pregnant Arab women’s emotional distress and feelings of the absence of social support during the pandemic [39].

The current study also found that, among the Jewish majority, parenthood moderated the association between COVID-19-related stress and psychological distress; however, this did not apply for the Palestinian minority. In other words, during the pandemic, parenthood improved individual resilience, as expressed through reduced psychological distress, for the Jewish majority only, not the Palestinian minority. As previous studies have shown, parenthood can reduce psychological distress if social support is available [31,40]. Thus, as the Palestinian minority were denied access to their core social support systems, represented by families and communities [19], parenthood did not act as a promoter of resilience for this group. While among the Jewish majority, who could perceive the government as a support system [28], parents reported less distress, indicating that parenthood had an impact on resilience. This finding clarifies the relevance of minority versus majority categorization when seeking to understand the effect of parenthood as a resilience factor. This highlights that the same factor (in this case, parenthood) can have different resilience effects on groups from different locations in the social structure (e.g., majority vs. minority). Therefore, when addressing multi-dimensional worldwide events such as pandemics, which impact all populations, it is important to note that not all groups are affected in the same way. During such events, public health experts and policy makers should apply cultural sensitivity and pay extra attention to discriminated groups with less social capital, such as minorities.

This study has several limitations. First, it used a cross-sectional design, which, although sufficient for collecting data during real-time crisis events, could only present the participants’ COVID-19-related stress and psychological distress at the time of data collection (approximately 2 months after the initial implementation of the pandemic restrictions in Israel). Therefore, this research method does not provide information on the development of the participants’ psychological distress over time (e.g., their psychological distress before the onset of the pandemic or changes over time). Since psychological distress is not static but temporally dynamic, future studies should monitor the levels of distress over several time points. For example, it is possible that, as the crisis continued, the minority and majority groups’ levels of psychological distress deteriorated further (as the situation worsened or through cumulative distress); alternatively, the groups may have developed resilience over time. These changes could be explored in a longitudinal study. Additionally, the chosen sampling method, although common, is also characterized by bias in favor of a population with relatively high technological literacy and who is available for online surveys. This aspect should also be taken into account when interpreting the findings.

A second limitation is that this study did not address diversity within the two groups. Both the Palestinian minority and Jewish majority are composed of people from various subgroups, such as different races/ethnicities, nativity statuses, religion and beliefs, occupations, and social classes. These differences may cause them to experience COVID-19 and the accompanying circumstances differently, and their COVID-19-related stress and psychological distress could also vary accordingly. Thus, to further understand how circumstances and social locations act as risk factors or protective and resilience factors, future studies could explore the differences within the two groups.

A third limitation is that the current study does not address the participants’ prior health status. As minorities in general, and the Palestinian group in particular, suffer from more health problems compared with majorities [27], they may experience COVID-19 and the accompanying circumstances differently, and their COVID-19-related stress and psychological distress could also vary accordingly. Thus, future studies could also explore this difference between the two groups.

## 5. Conclusions

The current study introduces the socio-ecological model as a framework for conceptualizing the differences between minorities and majorities, while reconfirming the differences between the Palestinian minority and the Jewish majority in Israel regarding COVID-19-related distress. The results emphasize the significance of socio-cultural context for understanding the differences between population groups with respect to distress and resilience during pandemic events. Although the COVID-19 pandemic has affected the general population in Israel across all social groups, and although the same instructions and restrictions have been administered to all citizens, the psychological impact of these instructions has varied across population groups. For the Palestinian minority, which had a higher a priori risk of psychological distress (as a minority group), the government restrictions worsened their distress (because of cultural and historical differences). This situation emphasizes the need for cultural sensitivity and cultural awareness when issuing instructions in such circumstances. Different groups may be provided with specialized instructions according to their cultural contexts. In circumstances where different instructions cannot be applied, culturally sensitive implementation of the instructions should be considered, along with the provision of resources that can help groups cope with the consequences and ensure their mental health. This can be accomplished through public health messaging, professional practice, and service configurations. Such conclusions are important for strategies dealing with future pandemics as, according to health care professionals, COVID-19 is not the last epidemic that the world will have to deal with.

In addition, this study conceptualized parenthood as a moderator of distress from a socio-ecological perspective. In other words, cultural context and social status (minority vs. majority) seem to be factors that determine the association between parenthood and resilience, a finding that needs to be studied further.

## Figures and Tables

**Table 1:** Sample characteristics.

Demographic Measures	Full Sample N = 1943
Ethnicity	Palestinian: 28.40% (*n* = 552) Jewish: 71.59% (*n* = 1391)
Age	M = 40.38, SD = 13.77
Years of education	M = 14.63, SD = 2.50
Gender	Female: 54.86% (*n* = 1066) Male: 45.13% (*n* = 877)
Residence	Urban: 75.14% (*n* = 1460) Rural: 24.85% (*n* = 483)
Impact of COVID-19 on employment status	Still working: 62.12% (*n* = 1207) Not working: 37.87% (*n* = 736)
Impact of COVID-19 on economic status	Income affected: 64.43% (*n* = 1252) Income not affected: 35.56% (*n* = 691)
Parenthood	Have children: 67.21% (*n* = 1306) Do not have children: 32.78% (*n* = 637)
Distress (DASS Total)	M = 11.53, SD = 12.53
COVID-19-related stress	M = 32.72, SD = 8.16

COVID-19: Coronavirus disease 2019; DASS: Depression, Anxiety, and Stress Scale.

**Table 2:** Moderation model.

Predictor	B	t	CI
Constant	15.34	17.85 ***	13.71, 16.90
COVID-19-related stress	0.57	16.60 ***	0.49, 0.66
Parenthood (1 = having children)	−1.30	−4.04 ***	−1.98, −0.56
Ethnicity (1 = Palestinian)	2.32	7.43 ***	1.62, 3.01
Age	−0.06	−3.21 **	−0.10, −0.03
Gender (1 = female)	0.33	1.36	−0.14, 0.80
Residence (1 = urban)	−0.35	−1.26	−0.95, 0.23
Impact of COVID-19 on employment status (1 = not working)	0.61	2.43 *	0.11, 1.11
Impact of COVID-19 on economic status (1 = income affected)	0.59	2.33 *	0.14, 1.05
Parenthood × COVID-19-related stress	0.002	0.08	−0.08, 0.08
Ethnicity × COVID-19-related stress	0.09	2.83 **	0.01, 0.18
Ethnicity × parenthood	0.32	1.10	−0.33, 1.02
Ethnicity × parenthood × COVID-19-related stress	0.09	2.65 **	0.005, 0.17

*F*(12, 1892) = 66.50, *p* < 0.001, R^2^ = 29.67%. *** *p* < 0.001, ** *p* < 0.01, * *p* < 0.05.

## Data Availability

The data presented in this study are available on request from the corresponding author.
